# Environmental Occurrence, Toxicity Concerns, Degradation Behavior, and Plant Resistance of Glufosinate-Ammonium

**DOI:** 10.3390/jox16060178

**Published:** 2026-09-22

**Authors:** Zhihao Wen, Hui Liu, Jiayi Zhang, Tianyue Wang, Yaohua Huang, Mohamed A. Ghorab, Shaohua Chen, Yonglin Liao

**Affiliations:** 1 Integrative Microbiology Research Centre, South China Agricultural University, Guangzhou 510642, China; 2 Wildlife Toxicology Laboratory, Department of Animal Science, Institute for Integrative Toxicology (IIT), Michigan State University, East Lansing, MI 48824, USA; 3 Guangdong Provincial Key Laboratory of High Technology for Plant Protection, Institute of Plant Protection, Guangdong Academy of Agricultural Sciences, Guangzhou 510640, China

**Keywords:** glufosinate-ammonium, herbicide, degradation behavior, toxicity, plant resistance

## Abstract

The market for traditional non-selective herbicides has undergone dramatic changes over the past decade. As an emerging herbicide, glufosinate-ammonium’s market share has been steadily increasing. However, with its widespread use, glufosinate-ammonium residues have been detected in rivers and agricultural products in many regions around the world. Furthermore, various toxicological studies have confirmed that glufosinate-ammonium has toxic effects on non-target organisms; therefore, its hazards should not be underestimated. To date, comprehensive reviews on the toxicity, degradation behavior, and resistance status of glufosinate-ammonium remain scarce. Herein, this article focuses on describing the toxicological properties and degradation behavior of glufosinate-ammonium and deeply explores the resistance mechanisms of plants to glufosinate-ammonium. This review deepens our understanding of the degradation and resistance mechanisms of glufosinate-ammonium and provides a theoretical basis and practical reference for the remediation of glufosinate-ammonium-contaminated environments.

## 1. Introduction

With the rapid development of agricultural modernization, the pursuit of efficiency has become a priority. The use of herbicides significantly reduces both labor and economic costs, and with the rising expense of manual labor, herbicides have seen increasingly widespread application. However, their excessive use has led to severe damage to both the ecological environment and soil health [1]. As an agricultural input, herbicides play a key role in controlling weeds and ensuring crop yields, and their use can reduce crop losses by 20% to 50% [2]. Currently, herbicides account for 47.5% of global pesticide use, primarily due to the expanding scope of herbicide application for weed control in agriculture [3]. Overall, herbicide consumption constitutes the largest proportion of global pesticide usage, making it one of the most critical pesticide categories.

Glufosinate-ammonium [2-Amino-4-[hydroxy(methyl)phosphoryl]butanoic acid ammoniate] is a broad-spectrum non-selective herbicidal agent successfully developed by Bayer AG in the 1980s [4]. It is a chiral molecule that typically exists as a racemic mixture of the L- and D-isomers, but only the L-isomer exhibits herbicidal activity [5,6]. Glufosinate-ammonium is a phosphonate belonging to the organophosphorus chemical family [6]. The physicochemical properties of glufosinate-ammonium are summarized in Table 1 [7]. In recent years, the production and consumption of glufosinate-ammonium have increased significantly; in 2023, global production capacity reached 121,800 metric tons, showing rapid year-over-year growth [8,9]. As a glutamine synthetase inhibitor with partial systemic activity, its toxicological mechanism involves inhibiting plant glutamine synthetase, which disrupts nitrogen metabolism, causes ammonium accumulation, and leads to chloroplast disintegration [10]. These processes impair photosynthesis, ultimately resulting in plant death [11]. The herbicidal mechanism of glufosinate-ammonium is shown in Figure 1.

Glufosinate-ammonium contains a chiral center and exists in two optical isomers, L-form and D-form. Most of the glufosinate-ammonium currently on the market is a racemic mixture containing equal amounts of D-glufosinate-ammonium and L-glufosinate-ammonium. Due to the chiral selectivity of glufosinate-ammonium, L-glufosinate-ammonium exhibits significant differences from the racemic mixture in terms of biological activity, toxicity, and environmental behavior; for example, L-glufosinate-ammonium has stronger herbicidal activity but is also more toxic to non-target organisms [12,13]. Studies have shown that compared with D-glufosinate-ammonium, only L-glufosinate-ammonium exhibits herbicidal activity [14,15].

In 1999, a report by the Joint FAO/WHO Expert Meeting on Pesticide Residues (JMPR) noted that the three main metabolites of glufosinate-ammonium—3-methylphosphinico-propionic acid (MPP), *N*-acetyl-glufosinate (NAG), and 2-methylphosphinico-propionic acid (MPA)—are all toxic substances (their structures are shown in Figure 2) [16]. The European Union, Japan, and the Codex Alimentarius Commission (CAC) define glufosinate-ammonium residues as the sum of glufosinate and its salts, MPP, and NAG, expressed as glufosinate-ammonium [17,18,19]. However, due to their ionic nature, low volatility, low molecular weight, and lack of easily detectable chemical groups, it is difficult to determine glufosinate-ammonium and its metabolites at low concentrations. Therefore, most glufosinate residues in the environment and food are detected through derivatization [20,21,22].

Due to the large-scale global use of herbicides, toxicological studies on herbicides have been conducted in recent years. These studies have found that herbicides present in natural ecosystems can cause significant ecological changes, such as the loss of soil fertility, and in severe cases, pose risks to human and animal health [23]. Additionally, herbicides can seriously threaten ecosystem safety through food chain contamination and groundwater pollution [24]. In addition to its herbicidal activity, glufosinate-ammonium also exhibits insecticidal and fungicidal activity and can be mixed with insecticides and other products to effectively prevent and control crop pests and diseases [16,17,19]. It has significantly contributed to increasing crop yields of grains, fruits, and vegetables while protecting plants from pests and diseases. Despite generating remarkable agricultural benefits, its use has also brought adverse environmental and human health consequences. Glufosinate-ammonium decomposes rapidly in soil with minimal residue, yet it may adversely affect aquatic environments. Due to its high water solubility (1370 g/L), it readily transfers into water bodies after agricultural application, causing water pollution. According to a report by the European Food Safety Authority (EFSA), MPP exhibits high mobility in water, while MPA exhibits high to moderate mobility in water; furthermore, MPP poses a risk of contaminating groundwater in vulnerable areas. MPP and MPA exhibit low to moderate persistence in soil. If most of the crop remains in the field after harvest, its environmental impact cannot be estimated [25,26]. Therefore, the potential ecological risks of glufosinate-ammonium warrant significant attention and caution.

Herbicide remediation mainly includes physical, chemical, and biological decomposition [27]. In natural environments, these herbicides can accumulate to high concentrations, posing life-threatening risks to humans and animals. Although physical and chemical decomposition methods are effective in removing residues, their high costs and tendency to cause secondary environmental pollution limit their practical application and development [28]. Therefore, microbial decomposition stands out as one of the safest and most effective strategies for controlling xenobiotic contamination [29,30,31]. So far, various microorganisms that can effectively degrade glufosinate-ammonium residues have been isolated, including bacteria and algae, and they play a crucial role in the degradation process [8,9,32].

However, there remains a notable lack of comprehensive reviews addressing the degradation mechanisms and pathways of glufosinate-ammonium. This gap hinders the development of efficient biological restoration strategies. For this reason, this review systematically summarizes: (1) the fate and residues of glufosinate-ammonium in the environment; (2) the toxic effects of glufosinate-ammonium on ecosystems; (3) the metabolic pathways and degradation mechanisms of glufosinate-ammonium; and (4) the molecular mechanisms of plant resistance to glufosinate-ammonium.

## 2. The Fate and Residues of Glufosinate-Ammonium in the Environment

The use of herbicides is an important part of modern agricultural production worldwide [33]. In recent years, with the steady increase in the number of transgenic plant varieties resistant to glufosinate-ammonium and the widespread adoption of transgenic plants, the use of this herbicide has been rising year by year. Consequently, this has also driven the growth of the glufosinate-ammonium market, resulting in year-over-year increases in sales [6]. However, field applications have shown that the continuous use of this compound has also caused environmental hazards, including soil residues of pesticides, migration in water bodies, and toxicity to non-target organisms. For example, Thompson et al. analyzed 200 honey samples from western Canada, and their findings showed that glufosinate-ammonium was detected in 125 of the samples, with the highest concentration reaching 33.0 μg/kg [6]. All these factors pose direct threats to ecosystem health and public health.

It is reported that glufosinate-ammonium can also enter the soil through ways such as atmospheric deposition, irrigation water, and residues of animals and plants [6,34]. Currently, the vast majority of glufosinate-ammonium applied via field spraying can be eliminated through microbial activity and precipitation; the portion that remains in plants can enter the soil after the leaves fall, where some of it is degraded by microorganisms [35,36]. However, due to its high water solubility, the remaining glufosinate-ammonium easily enters water bodies, causing pollution to the aquatic environment. Concentrations of glufosinate-ammonium as high as 0.72 μg/L have been detected in rivers in northern Italy, exceeding the European Union’s maximum permissible concentration for pesticides in river water (0.1 μg/L) [37]. Samples taken from surface water in farmland across 17 provinces and municipalities in China revealed maximum glufosinate-ammonium concentrations of up to 13.15 μg/L [38]. According to an environmental concentration assessment conducted in Canada, glufosinate-ammonium concentrations in the environment are estimated to reach 1 mg/L [39].

The circulation and flow of glufosinate-ammonium in the natural environment start from farmlands and form a multi-media migration cycle along the “soil–water–organism” pathway. After application in farmlands, most of the glufosinate-ammonium first remains in the surface soil: a part is adsorbed by soil particles and temporarily fixed, while the other part penetrates downward through soil leaching and enters the groundwater system. During rainfall or irrigation, the adsorbed glufosinate-ammonium flows into surface water along with farmland runoff, becoming the main source of glufosinate-ammonium in the aquatic environment [38,40].

After entering the water body, part of the glufosinate-ammonium will be absorbed by aquatic plants or spread to more distant water areas with water flow; another part may settle into the bottom sediment, come into contact with microorganisms in the sediment, and undergo degradation. Glufosinate-ammonium in soil and water can also enter the food chain through biological migration: after crops absorb the residues in the soil, they enter the livestock and poultry breeding link through harvesting or directly affect humans through food crops; after aquatic organisms take in glufosinate-ammonium from water, they are preyed on by organisms of higher trophic levels, realizing cross-trophic level circulation [34,41].

According to a bioaccumulation study of glufosinate-ammonium in zebrafish, after 14 days of exposure to 2 mg/L glufosinate-ammonium, the bioaccumulation of both D-glufosinate-ammonium and L-glufosinate-ammonium in zebrafish increased with exposure time. The concentration of D-glufosinate-ammonium was 0.036 mg/kg on day 1 and increased to 0.17 mg/kg by day 14, while that of L-glufosinate-ammonium increased from 0.014 mg/kg on day 1 to 0.21 mg/kg by day 14. In the 10 mg/L glufosinate-ammonium concentration group, the concentrations of L-glufosinate-ammonium and D-glufosinate-ammonium increased from initial levels of 0.12 mg/kg and 0.11 mg/kg to 0.85 mg/kg and 0.75 mg/kg, respectively; throughout the experiment, the concentration of L-glufosinate-ammonium consistently exceeded that of D-glufosinate-ammonium, demonstrating that L-glufosinate-ammonium exhibits a stronger bioaccumulation effect [42]. In addition, due to the potential health risks associated with glufosinate-ammonium, there is growing concern about exposure to this pesticide and its metabolites. After humans ingest glufosinate-ammonium through food and water, it is primarily excreted in feces and urine; the Joint Meeting on Pesticide Residues (JMPR) has set the acute reference dose for glufosinate at 0.01 mg/kg·bw [43]. Therefore, glufosinate-ammonium can cause harm to humans and animals through food and drinking water. The residues of glufosinate-ammonium in the environment are illustrated in Figure 3.

The use of herbicides is an important means of increasing agricultural output and farmers’ income. With the continuous increase in the number of herbicide varieties, the expansion of cultivated crop areas, and the growing amount of herbicide application, a series of environmental issues arising from herbicide use have gradually drawn attention [44,45]. Glufosinate-ammonium, as one of the most commonly used organophosphorus herbicides, has consistently been a focus of concern regarding the environmental problems it causes during application. Since glufosinate-ammonium was developed in the 1980s, its usage has been on the rise. There have been reports indicating that residues of this pesticide have been detected in various water environments around the world [46]. Studies have found that glufosinate-ammonium can enter water bodies via runoff, and residues have been detected in runoff from oil palm plantations in tropical rainforest regions [47]. In 10 provinces in China, including Gansu, Guangxi, and Hebei, glufosinate-ammonium concentrations in surface water ranged from 2.38 to 13.15 μg/L [38].

Moreover, glufosinate-ammonium residues in water bodies can exert certain impacts on aquatic plants, animals, and other organisms. As producers and consumers in the aquatic ecosystem, algae and fish exhibit various physiological responses to changes in the water environment. With the improvement in human living standards, people are paying more attention to pesticide residues in food and the environment. Currently, countries around the world have established maximum residue limits (MRLs) for glufosinate-ammonium in food. For example, the Codex Alimentarius Commission (CAC) sets the limit for glufosinate-ammonium at 0.02–8 mg/kg [48]; the United States and Japan set it at 0.05–6 mg/kg [16]; Canada sets it at 0.1–2 mg/kg [17]; and the European Union sets it at 0.1–5 mg/kg [49]. The MRLs of glufosinate-ammonium in different countries are shown in Table 2 [50,51,52,53,54,55,56].

The residual glufosinate-ammonium in the environment causes multi-dimensional impacts on soil, water bodies, and ecosystems. Residual glufosinate-ammonium in soil inhibits the activity of soil microorganisms and disrupts the soil carbon–nitrogen cycle. For instance, it reduces the activity of key enzymes such as urease and phosphatase, leading to a decline in soil fertility. Long-term accumulation may also alter the structure of soil microbial communities, affecting the stability of soil ecological functions [57].

However, the aforementioned research methods have certain limitations. Environmental residues of glufosinate-ammonium vary significantly among different countries. For example, Canada’s environmental assessment model predicts that glufosinate-ammonium concentrations in the environment could reach 1 mg/L, which is 2–3 orders of magnitude higher than results from other countries; there is a stark discrepancy between the model-predicted concentrations and actual monitoring results. Furthermore, bioaccumulation tests were conducted under indoor laboratory conditions, which differ significantly from real-world field environments. The exposure concentrations set for zebrafish bioaccumulation tests—2 mg/L and 10 mg/L—are far higher than globally measured levels in surface water. Therefore, it cannot be directly proven that bioaccumulation effects of the same magnitude would occur under field conditions, and the results are subject to uncertainty. In summary, due to the long-term use of glufosinate-ammonium, its residues have been detected in water and soil systems in many countries around the world, posing significant risks to non-target organisms and soil quality. Therefore, we encourage the proper management of glufosinate-ammonium to mitigate its potential risks to the environment and human health.

## 3. Abiotic Degradation of Glufosinate-Ammonium

Pesticides have made tremendous contributions to global food production. However, with the increasing annual usage of pesticides, they are prone to entering the aquatic environment through various pathways such as drift, rainwater scouring, and surface runoff, thereby disrupting the aquatic ecosystem, causing severe harm to aquatic organisms, and posing a threat to human health [58]. The hydrolysis and photolysis of pesticides in the aquatic environment are common phenomena of chemical degradation, serving as one of the primary forms of abiotic degradation and crucial indicators for evaluating the residual characteristics of pesticides in water bodies [59].

Previous experiments have shown that the half-lives of D-glufosinate-ammonium and L-glufosinate-ammonium are not the same. For example, experiments have shown that under sterile soil conditions, the half-lives of the D- and L-enantiomers of glufosinate-ammonium are approximately 12.2 days and 8.0 days, respectively. In addition, after 100 days in water, the concentration of the L-enantiomer of glufosinate-ammonium decreased from 4.99 mg/L to 4.20 mg/L, with a degradation rate of 21.4%, while the concentration of the D-enantiomer decreased from 4.94 mg/L to 4.20 mg/L, with a degradation rate of 15.0%. No interconversion between the enantiomers was observed during the degradation process [60].

Laboratory studies have found that the half-life of L-glufosinate-ammonium in soil samples from Daxing, Beijing; Jinan, Shandong; Changsha, Hunan; and Nanning, Guangxi, ranges from 3.40 to 12.20 days [60]. Chen et al. found that the half-life of glufosinate-ammonium varies with room temperature and season. At a room temperature of 4 °C, the half-life is 21.00 days; at 25 °C, it shortens to 14.40 days. The half-lives of glufosinate in soil during summer and fall are 2.50 days and 6.60 days, respectively [61]. In addition, the degradation rate of glufosinate-ammonium is correlated with soil pH, light intensity, soil organic matter (SOM), clay content, and cation exchange capacity (CEC) [62].

Under weakly acidic conditions, the hydrolysis half-life of glufosinate is slightly shorter than its degradation rate under weakly alkaline conditions, while the degradation rate of glufosinate-ammonium is the slowest under neutral conditions. In buffer solutions with pH values of 5.0, 6.9, and 9.3, the hydrolysis half-life of glufosinate-ammonium reaches 400 days. The results of experiments conducted at the enantiomer level show that the half-lives of the D- and L-glufosinate-ammonium enantiomers in sterile buffer solutions with pH values of 5, 7, and 9 can exceed 400 days. In naturally sourced pond water, L-glufosinate-ammonium degrades slightly faster than D-glufosinate-ammonium. At 100 days, the degradation rate of L-glufosinate-ammonium is 21.4%, while that of D-glufosinate-ammonium is 15% [63].

In addition, glufosinate-ammonium dissolved in gravel pit surface water was exposed to ultraviolet (UV) radiation at 25 °C for 118 h (equivalent to 33 days of sunlight exposure). Under this condition, glufosinate-ammonium barely degraded, with only 3–5% of it converted into 3-methylphosphinicopropionic acid (MPP). It can be concluded that the photodegradation of glufosinate in natural aquatic environments is a relatively inefficient degradation pathway, which enables the compound to persist in such systems over the long term and thus induces toxic effects on aquatic organisms [6].

Hydrolysis and photolysis of herbicides in aquatic environments are common phenomena in the chemical degradation of herbicides; they are among the primary forms of abiotic degradation and serve as important indicators for evaluating the residue characteristics of herbicides in water bodies. The aforementioned studies indicate that the degradation rate of L-glufosinate-ammonium is generally faster than that of D-glufosinate-ammonium; environmental factors can influence the degradation rate of glufosinate-ammonium.

However, the pH gradients in the above experiments were too narrow, and there is insufficient evidence to support the conclusion that the degradation rate of glufosinate-ammonium is slowest under neutral conditions. Furthermore, there are significant variations in the soil half-life of glufosinate-ammonium across different experiments; it is speculated that this may be due to the fact that the relative contributions of other factors—such as regional soil differences, season, and temperature—have not yet been quantitatively separated. In summary, the hydrolysis and photolysis of glufosinate-ammonium in aquatic environments proceed slowly, suggesting that it is prone to becoming a persistent pollutant. Therefore, we need to implement appropriate measures to regulate the application of glufosinate-ammonium.

## 4. Biotic Degradation of Glufosinate-Ammonium

With regard to pesticide residues in soil and water, microbial degradation is one of the primary pathways for the metabolism of these residues. Factors influencing degradation include the physicochemical properties of the pesticides, soil conditions, environmental conditions, and the diversity of soil microorganisms [64,65]. Microbial degradation of pesticides offers the advantages of a wide range of applications and diverse metabolic pathways; moreover, compared to physical and chemical degradation methods, it is safer, more environmentally friendly, and more effective [66]. Therefore, it holds great promise for further research and application.

Studies have shown that microbial degradation plays a significant role in the enantioselective degradation of chiral compounds in soil [67,68,69]; therefore, soil microbial activity can directly influence the degradation rate of glufosinate-ammonium. For example, under non-sterile conditions, the half-lives of the L- and D-enantiomers of glufosinate range from 1.4 to 4.3 days, which is significantly shorter than under sterile soil conditions [70,71,72]. Furthermore, the use of glufosinate-ammonium has a certain degree of impact on soil physicochemical properties, enzyme activity, and microorganisms. Glufosinate-ammonium residues can alter soil microbial diversity, and their metabolites may interact with soil organic matter and heavy metals, thereby indirectly affecting microbial activity [73]. Research has found that, compared to mechanical weeding, glufosinate-ammonium not only removes weeds but also alters the nutrient composition of the grapevine rhizosphere and significantly affects the formation of rhizosphere microbial communities [57]. Pattison et al. established two treatments—mowed and unmowed—in banana fields. In each of the two treatment plots, they applied no glufosinate-ammonium, the recommended dose (1 kg a.i./ha), or twice the recommended dose (2 kg a.i./ha) of glufosinate-ammonium. The results indicated that applying glufosinate-ammonium (in the presence of weeds) did not affect soil bacterial and fungal diversity; however, applying glufosinate-ammonium alone after mowing the weeds indirectly affected soil microbial activity and function [74]. Therefore, it is hypothesized that repeated, high-dose applications of glufosinate-ammonium may affect soil microorganisms.

Wang et al. isolated a bacterial strain capable of efficiently degrading glufosinate-ammonium, which was identified as *Rhodococcus gordoniae*. Degradation tests showed that when the incubation temperature was 35 °C, the soil concentration of glufosinate-ammonium was 800 mg/kg, the pH was 6.38, and the inoculation rate was 1% (*v*/*w*). Under these conditions, this strain achieved a glufosinate-ammonium degradation rate of up to 95%, demonstrating significant biotic degradation potential. Further research has shown that jasmonic acid, a type of plant hormone, attracts *R. gordoniae* and promotes its growth and metabolic activity, thereby effectively degrading glufosinate-ammonium residues in the soil [32].

Liu et al. investigated the degradation efficiency and molecular mechanisms of glufosinate-ammonium by *Dunaliella salina*. The study found that *D. salina* exhibited a maximum tolerance concentration of 0.8 mg/mL for glufosinate-ammonium and achieved a degradation efficiency of 91.74% for glufosinate-ammonium in natural seawater within 3 days. The study also revealed that *D. salina* can achieve highly efficient degradation of glufosinate-ammonium through multiple biological responses, including phosphorus utilization and acetyltransferase-related stress responses [9].

Currently, there has been new progress in biotic degradation of glufosinate-ammonium by *Klebsiella pneumoniae* OC54 and plants. In transgenic plants, the expression of the *PAT* gene results in the detoxification of glufosinate-ammonium into less toxic NAG through acetylation. This substrate is more mobile within the cell and is further metabolized into less toxic derivatives, such as MHB/MPB, MPP, and subsequently MPA, as indicated by the bold blue arrows. In non-transgenic plants, OC54-mediated detoxification and metabolic support primarily reduce glufosinate-ammonium concentrations through adsorption, sequestration, and biofilm formation, physically limit herbicide uptake, and enhance buffering capacity and reduce bioavailability, as indicated by the dashed green arrows [8]. The biotic degradation pathway of glufosinate-ammonium is illustrated in Figure 4.

## 5. Toxic Effects of Glufosinate-Ammonium on Ecosystems

Glufosinate-ammonium has many advantages, including a broad spectrum of weed control, low toxicity, high herbicidal activity, and excellent environmental adaptability. As a result, it is widely used in non-cultivated areas to control various annual and perennial grasses and broadleaf weeds. In recent years, as highly toxic herbicides such as paraquat have been successively banned [75], the use of glufosinate-ammonium has continued to expand. However, glufosinate-ammonium has strong water solubility and persistent activity, and after being applied in the field, it tends to migrate and spread, which can lead to contamination of surface water, groundwater, and soil, making it a type of pseudo-persistent pollutant and posing potential safety risks to the ecological environment and human health.

### 5.1. Toxic Effects of Glufosinate-Ammonium on Non-Weed Plants

In experiments conducted to evaluate the safety of glufosinate-ammonium on cash crops, the root weight and plant weight of wheat seedlings exposed to glufosinate-ammonium both showed a significant decrease compared to the control group, and the content of the key metabolite catechin was also significantly lower than that of the control group [76].

A toxicological assessment of aquatic ecosystems has shown that glufosinate-ammonium has significant ecotoxicological effects on algal populations, which are primary producers. When *Microcystis aeruginosa* was exposed to environmentally relevant concentrations of glufosinate-ammonium, the algal cell count increased significantly at concentrations below 1 mg/L, indicating that low concentrations of glufosinate-ammonium have a certain growth-promoting effect on *Microcystis aeruginosa*. However, when the concentration exceeded 5 mg/L, the algal cell count decreased significantly and malondialdehyde levels and electrolyte leakage rates increased significantly, demonstrating that glufosinate-ammonium caused oxidative damage and reduced cell membrane integrity in *Microcystis aeruginosa* [77]. Furthermore, a metabolomics study using *Chlorella vulgaris* as the experimental subject showed that 12 h acute exposure significantly activated its antioxidant enzyme system, but mRNA expression levels of core photosynthetic genes were suppressed. This finding reveals that glufosinate-ammonium can disrupt the energy metabolism homeostasis of algae through a dual mechanism [78]. The above study confirms that glufosinate-ammonium poses potential ecological risks to non-target aquatic plant communities.

### 5.2. Toxic Effects of Glufosinate-Ammonium on Non-Target Animals

Glufosinate-ammonium can cause damage to the hepatopancreatic tissue of crayfish, induce oxidative stress in crayfish, and reduce non-specific immunity [79]. In addition, previous studies have shown that glufosinate-ammonium exposure is associated with a range of embryonic malformations, including yolk sac edema, caudal curvature, and spinal deformities [80]. Glufosinate-ammonium has been shown to induce liver damage in a zebrafish model, and inhibition of the Nrf2 pathway has been identified as a key mechanism underlying this adverse reaction [81]. Following acute exposure experiments using 3.75, 7.5, and 15 mg/L glufosinate-ammonium on tadpoles of the common toad (*Rhinella arenarum*), the red blood cell chromosomes of tadpoles in each treatment group exhibited varying degrees of damage [82].

The reproductive system of fish has a profound impact on their health and survival and plays a vital role in maintaining fish populations [83]. In recent years, as research on glufosinate-ammonium has garnered increased attention, a growing number of reports have confirmed that glufosinate-ammonium exhibits certain reproductive toxicity. Studies have shown that glufosinate-ammonium causes oxidative stress damage to the testes of male lizards (*Eremias argus*) and alters their sex hormone levels [84]. According to a report, glufosinate-ammonium primarily damages the epigenome and transcriptome of mouse sperm [85]. Mitochondrial respiratory efficiency in human sperm is also affected following exposure to glufosinate-ammonium in experiments [86]. These findings suggest that glufosinate-ammonium causes damage to male sperm and the mitochondria within the sperm. Table 3 lists the toxic effects of glufosinate-ammonium at different concentrations on non-target organisms. However, in the aforementioned experiments, the exposure concentrations of glufosinate-ammonium were mostly higher than the measured μg/L levels in natural surface water; therefore, extrapolating these results to field populations involves a high degree of uncertainty. Furthermore, natural water bodies contain organic matter and clay particles that adsorb glufosinate-ammonium, thereby reducing its effective concentration in the aqueous phase. In contrast, the free concentration of glufosinate-ammonium is higher in laboratory pure water systems; consequently, the effect concentrations obtained in the laboratory may underestimate the actual safety thresholds in the field.

In summary, the inappropriate handling of glufosinate-ammonium and potentially hazardous effects on non-target organisms have received considerable attention within the scope of public health.

## 6. The Molecular Mechanisms of Plant Resistance to Glufosinate-Ammonium

Up to now, a variety of weed species have been confirmed to have evolved resistance to glufosinate-ammonium. In 2009, researchers first detected glufosinate-ammonium resistance in populations of *Eleusine indica*, marking the first reported case of glufosinate-ammonium-resistant weeds worldwide [90]. Following the discovery in *Lolium perenne*, *Lolium rigidum* and *Lolium multiflorum* have also been successively found to exhibit glufosinate-ammonium resistance, and the resistance mechanisms of these weeds are equally unclear [91,92]. With the long-term and widespread use of herbicides, the phenomenon of weeds developing resistance to these chemicals has become increasingly common [93]. To date, 99 crop species, 154 dicotyledonous weed species, and 115 monocotyledonous weed species across 72 countries or regions have developed varying degrees of resistance to 166 herbicides with 21 different mechanisms [94]. The emergence and rapid spread of herbicide-resistant weeds have posed new challenges to weed management that relies on chemical herbicides and have become a serious threat to global agricultural production and food security. Under long-term selective pressure, weeds have evolved various mechanisms to counteract herbicides [95]. With the complete sequencing of numerous weed genomes, an increasing number of researchers are utilizing transcriptomic sequencing to uncover the underlying mechanisms of herbicide resistance in weeds [96,97,98].

### 6.1. Regulation of GS Gene Expression in Crops

GS serves as the targeted action site of glufosinate in plants. The elevated expression and activity of this enzyme can effectively strengthen plant stress resistance against glufosinate-ammonium. *Amaranthus palmeri* is a weed species with resistance to glufosinate-ammonium. Under glufosinate-ammonium stress, the expression level of *GS2* in the plant can be up-regulated by 190-fold, and the expression level of *GS1* can be increased to up to 17-fold the original level. The overexpression of glutamine synthetase is regarded as the dominant inducing factor for *A. palmeri* to develop glufosinate-ammonium resistance [99].

Further studies have demonstrated that the loss-of-function mutation of the *OsSPL10* gene located on chromosome 6 in the glufosinate-ammonium-resistant rice mutant *gar6-2* can significantly enhance the glufosinate-ammonium tolerance of the plant. After exposure to glufosinate-ammonium stress, compared with the wild-type rice, the *gar6-2* mutant exhibited the de-repression of *OsGS1;1* and *OsGS2* expression due to the functional loss of *OsSPL10*, which consequently led to a marked up-regulation in the transcription level of *OsGSs* (glutamine synthetase-encoding genes) and a corresponding enhancement in enzyme activity. This alteration effectively alleviated the ammonium toxicity induced by glufosinate-ammonium, ultimately improving the herbicide resistance of the plant. It is thus evident that the *OsSPL10* gene acts as a negative regulator in the regulatory pathway of rice glufosinate-ammonium resistance [100]. The resistance mechanism of *gar6-2* rice to glufosinate-ammonium is illustrated in Figure 5.

### 6.2. BAR/PAT Transgenic Plants

In the 1980s, researchers cloned the bialaphos resistance (*BAR*) gene and the phosphinothricin acetyltransferase (*PAT*) gene from the soil bacteria *Streptomyces hygroscopicus* and *Streptomyces viridochromogenes*, respectively. These two gene sequences share 86% homology, and both encode an acetyltransferase capable of converting glufosinate-ammonium into the non-toxic *N*-acetyl-glufosinate-ammonium [101,102]. Transferring the *BAR* gene or *PAT* gene into plants can confer resistance to glufosinate-ammonium. For example, when the *BAR* gene is transferred into corn, the corn can tolerate 3600 g of glufosinate-ammonium per hectare [103]. To date, these two genes have been successfully used to develop commercially available glufosinate-resistant crops such as rice, soybeans, and cotton [104,105]. *RePAT* protein was isolated from the bacterium *Rhodococcus* sp. strain YM12. This protein exhibits extremely high catalytic efficiency toward glufosinate-ammonium in vitro, and when the gene is transferred into the *japonica* rice variety Zhonghua11, it enables it to tolerate 5000 g of glufosinate-ammonium per hectare, which is 10 times the commercial application rate of glufosinate-ammonium [106].

### 6.3. Mutation and Modification of the GS

Mutating key sites in the *GS* gene to reduce its ability to bind glufosinate-ammonium can also increase plant resistance to glufosinate-ammonium. Research confirmed that a Ser59Gly mutation in the target enzyme of glufosinate-ammonium, cytoplasmic glutamine synthetase (*EiGS1-1*), is a key molecular mechanism underlying the target resistance of *Eleusine indica* to glufosinate-ammonium [107]. Analysis of soybean cells resistant to glufosinate-ammonium revealed a His249Tyr mutation in the *GS* gene; His249 is the binding site for glufosinate-ammonium; a mutation at this site reduces GS sensitivity to glufosinate-ammonium, thereby conferring resistance to glufosinate-ammonium on soybean cells [108]. Using the DNA shuffling technique, the researchers discovered that Arg295Lys mutation is the key site for *OsGS1* protein resistance to glufosinate-ammonium and that introducing the mutated *OsGS1* into both *Saccharomyces cerevisiae* and *Arabidopsis* resulted in increased resistance to glufosinate-ammonium [109]. Using CRISPR-Cas9 gene editing technology, researchers have successfully developed wheat resistant to nicosulfuron herbicide [110].

### 6.4. Non-Target-Site Resistance (NTSR) of Plants to Glufosinate-Ammonium

As an important type of herbicide resistance, non-target-site resistance has extremely complex regulatory mechanisms, which can be mainly summarized into three aspects: first, accelerating the degradation process of herbicides and decomposing intracellular herbicides into non-toxic or low-toxic derivatives through relevant enzymatic reactions; second, changing the absorption and transport mode of herbicides and improving resistance by reducing the absorption efficiency of herbicides by cells or enhancing the sequestration of herbicides in vacuoles; third, activating the antioxidant system of plant cells to alleviate the oxidative stress response induced by herbicides and enhance the tolerance of plants [111,112].

Compared with target-site resistance (TSR), there are significant differences in their resistance characteristics: TSR has a relatively narrow resistance spectrum and only acts on herbicides targeting specific proteins; in contrast, non-target-site resistance (NTSR) can exhibit unpredictable resistance responses to herbicides with various modes of action (including non-commercialized herbicides). Its unpredictability is specifically reflected in two aspects: the difference in resistance levels and the broad spectrum of herbicides with resistance [113]. Plant cytochrome P450 is a large family of enzymes, and many P450 enzymes possess the ability to degrade herbicides. For example, *CYP76B1* from *Helianthus tuberosus* and *CYP86A4* from *Arabidopsis* can degrade phenylurea herbicides and paraquat herbicides, respectively, turning them into harmless substances [114,115]. The mechanisms of non-target-site resistance to glufosinate-ammonium in plants have not yet been clarified.

At present, our current understanding of most glufosinate-resistant populations remains limited, so the mechanisms of glufosinate resistance require further study. Finally, although glufosinate resistance has not yet evolved in many species, different strategies should be considered to maintain the efficacy of this herbicide and slow the evolution of multiple herbicide resistance.

## 7. Conclusions

As the use of glufosinate-ammonium in agricultural management continues to increase, it has led to the release of residual pollutants into the environment. In particular, because it is difficult to hydrolyze and photodegrade, it easily becomes a persistent pollutant. As it moves through the food chain, it poses health risks to non-target organisms and severely impacts the ecosystem. These issues require urgent attention, and it is necessary to develop reasonable herbicide management strategies and remedial measures. Microbial degradation is one of the most effective and environmentally friendly methods for herbicide removal. While some microorganisms and algae with degradation capabilities have been identified, there remain significant gaps in our understanding based on current research. For example, most degradation parameters are derived from simplified laboratory conditions, which differ significantly from real field environments. Existing toxicological studies primarily involve short-term, high-concentration indoor exposure, which does not correspond to actual residue concentrations in the field, making it difficult to accurately assess ecological and health risks under real conditions. The number of highly efficient microbial degradation resources is limited; the key degradation genes and enzymes of most strains have not yet been fully characterized, and there is a lack of validation through field trials, making it difficult to apply them directly to actual environmental remediation. Furthermore, there is currently insufficient understanding of the evolution and spread patterns of glufosinate-ammonium-resistant weeds, which hinders the prevention and control of resistance in the field. Therefore, there are some important issues that require further study to refine the research direction for the future management of glufosinate-ammonium in agriculture.

(a) Conduct field trials to study the environmental behavior of glufosinate-ammonium and its metabolites and improve environmental risk prediction.
(b) Conduct long-term toxicity tests at actual environmental concentrations to supplement toxicity data for various organisms and refine ecological risk assessments.
(c) Identify additional degrading bacterial strains, and, through molecular biology techniques such as genomics, proteomics, and metabolomics, characterize and engineer the genes responsible for the degradation of glufosinate-ammonium, which is very significant for environmental remediation.
(d) Continuously monitor the occurrence of glufosinate-ammonium-resistant weeds in the field, elucidate the molecular mechanisms of resistance, develop integrated weed management strategies, and slow the evolution of resistance.

## Figures and Tables

**Figure 1:**
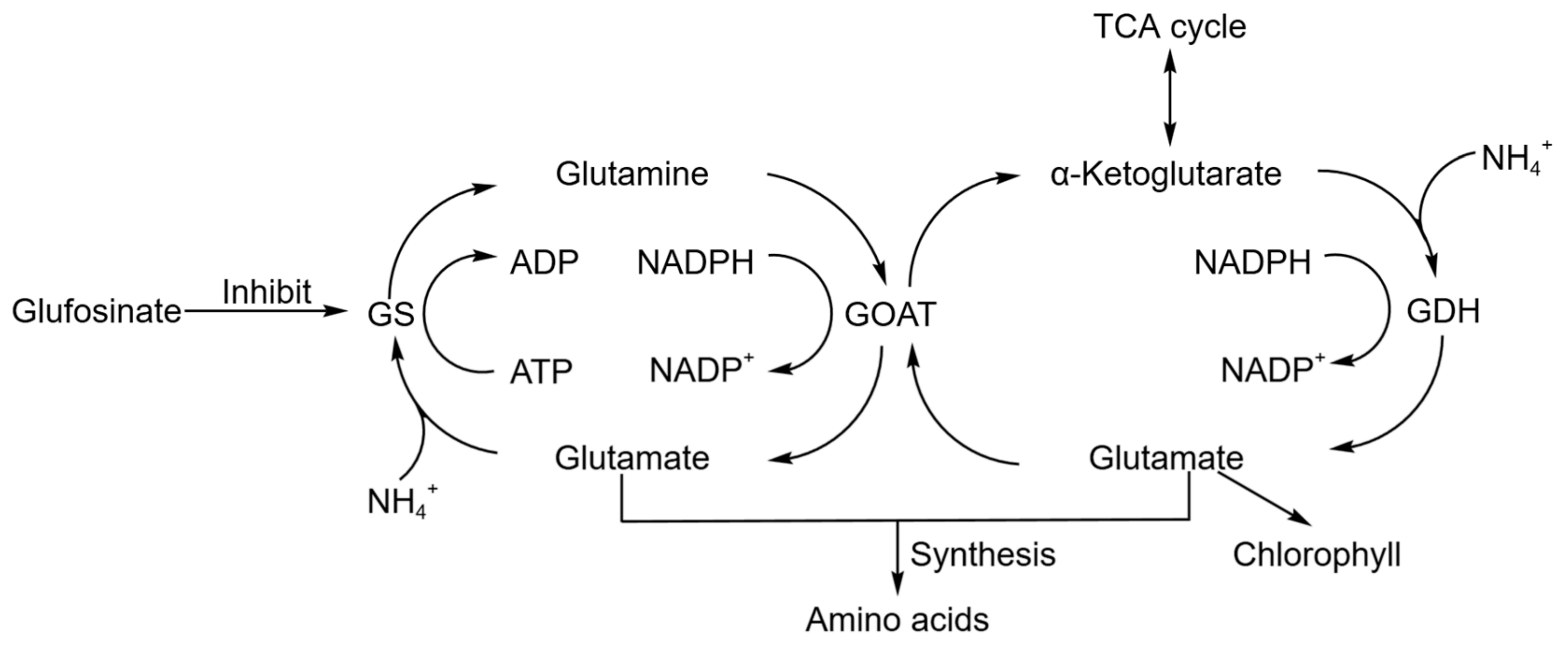
The herbicidal mechanism of glufosinate-ammonium [6,10]. GS: glutamine synthetase; GOAT: glutamate synthase; GDH: glutamate dehydrogenase; ADP: adenosine diphosphate; ATP: adenosine triphosphate; NADPH: nicotinamide adenine dinucleotide phosphate; NADP^+^: the oxidized form of NADPH; TCA cycle: tricarboxylic acid cycle. Arrow means the direction of chemical substance transformation.

**Figure 2:**
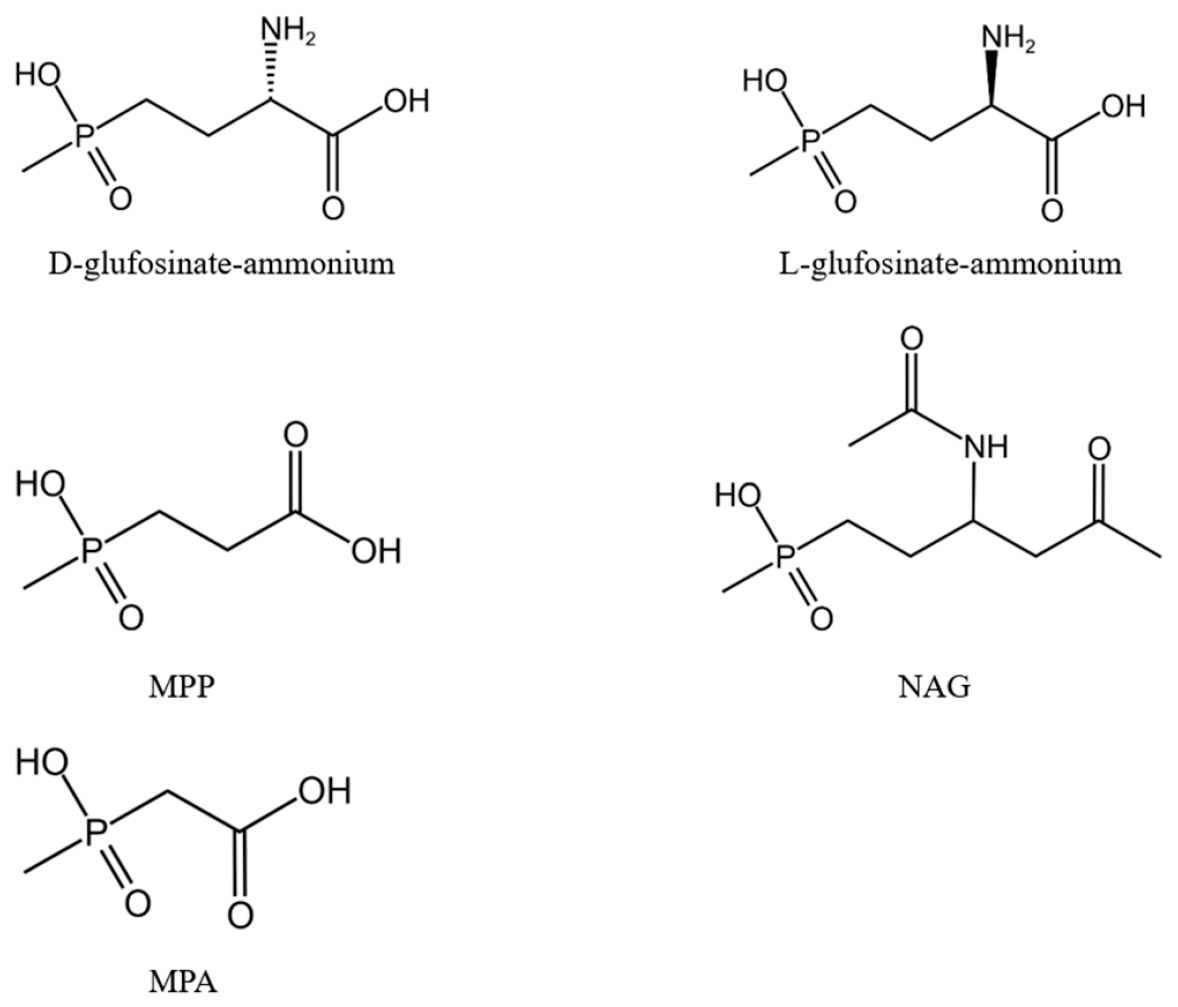
Structural formula of D-glufosinate-ammonium, L-glufosinate-ammonium, MPP, NAG and MPA. MPP: 3-methylphosphinico-propionic acid; NAG: *N*-acetyl-glufosinate; MPA: 2-methylphosphinico-propionic acid.

**Figure 3:**
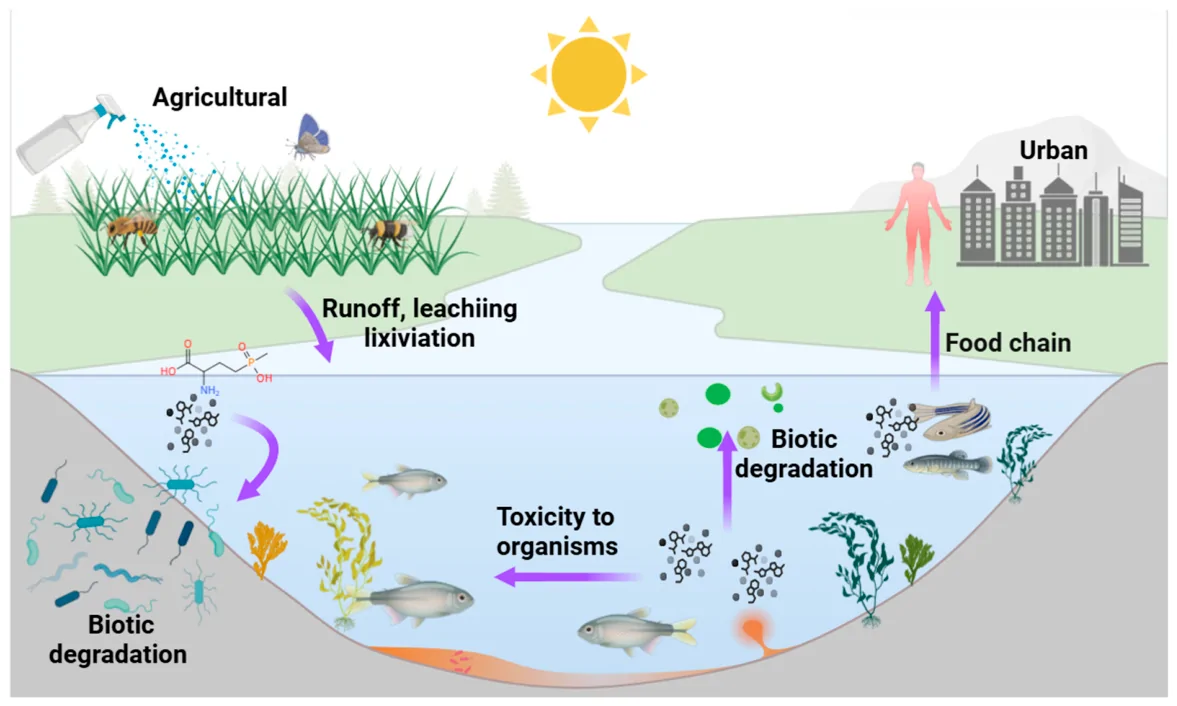
Environmental occurrence, toxicity, and degradation of glufosinate-ammonium.

**Figure 4:**
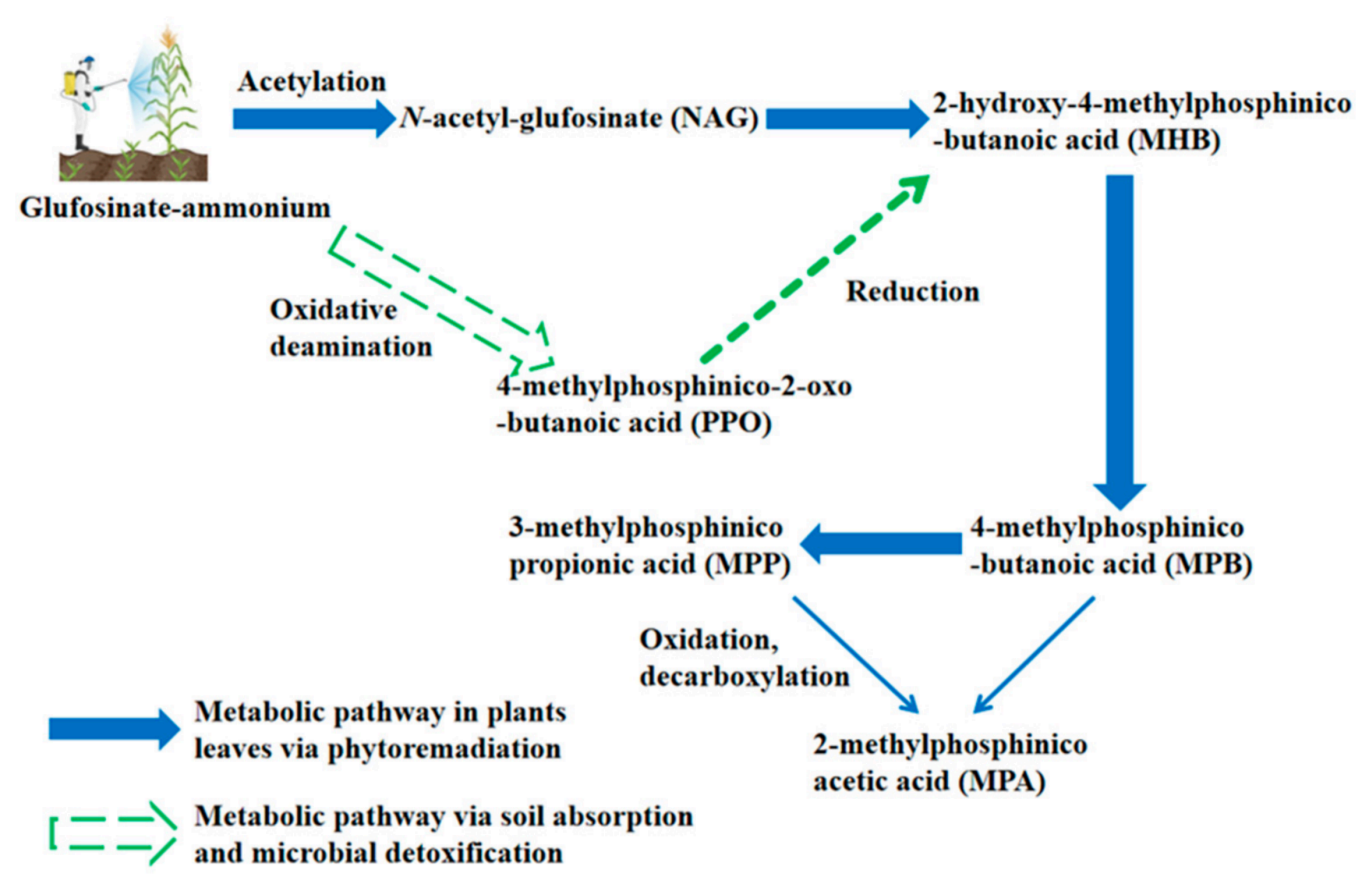
The microbial-assisted glufosinate-ammonium detoxification pathway and tolerance in plants [8].

**Figure 5:**
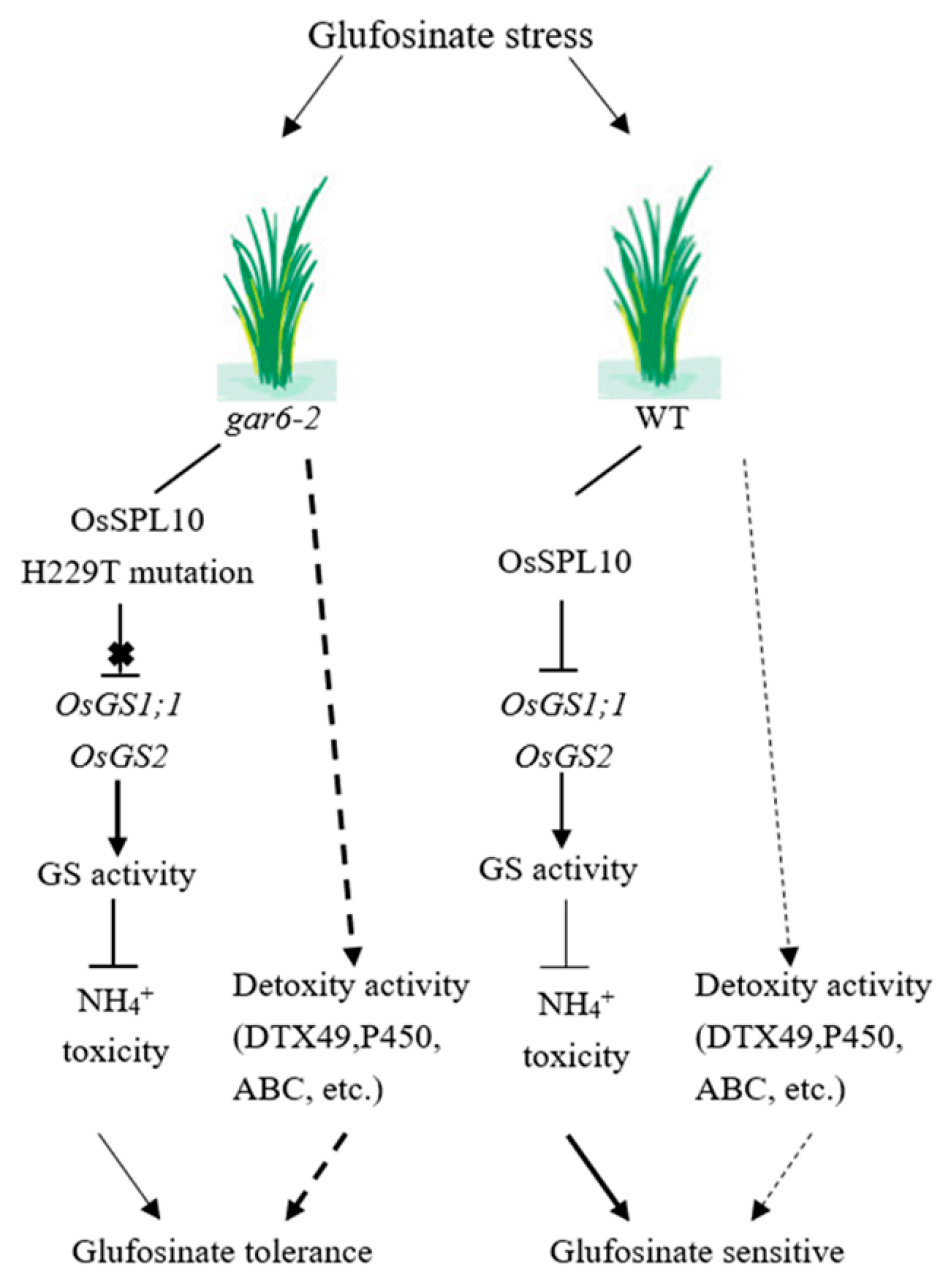
Working model for the molecular mechanism underlying glufosinate-ammonium resistance in *gar6-2* mutant rice [100]. Note: Solid lines indicate direct regulation of downstream genes or traits. Dashed lines indicate regulation (either direct or indirect) of downstream genes or traits or potential regulatory effects. Lines with arrows represent positive regulation, whereas lines with a transverse bar at the end denote negative regulation. The thicker the line, the stronger the regulatory intensity.

**Table 1:** Physicochemical properties of glufosinate-ammonium (all parameters are at 25 °C unless specified).

Physicochemical Properties	Value
Chemical formula	C_5_H_15_N2O_4_P
Relative molecular mass	198.157
CAS Registry Number	77182-82-2
ECHA number	278-636-5
CIPAC code numbers	437
Boiling point	519.1 °C
Melting point	210 °C
Flash point	100 °C
Appearance	White crystalline powder
Density	1.4 g/cm^3^
Acid dissociation constant	9.15 (at 20 °C)
Use type	Herbicide
Solubility (Water)	1370 g/L

**Table 2:** Comparison of maximum residue limits (MRLs) of glufosinate-ammonium on transgenic plants in different countries and regions.

Sample	MRLs of Glufosinate-Ammonium (mg/kg)							
	China	Codex Alimentarius Commission	USA	European Union	Japan	Canada	Australia	Korea
Soybean	2	2	2	2	2	2	2	2
Maize	0.1	0.1	0.2	0.1	0.1	0.2	0.2	0.05
Rape seed	1.5	1.5	0.4	1.5	5	3	5	0.3
Rape seed oil, crude	0.05	0.05	-	-	0.05	-	-	-
Cotton seed	5	5	15	5	5	3.5	3	3
Sugar beet	1.5	1.5	0.9	1.5	2	-	-	-
Sugar beet molasses	-	8	5	-	-	-	-	-

**Table 3:** Toxic effects of glufosinate-ammonium on different living systems.

Species	Concentration of Glufosinate-Ammonium	Effects	References
Zebrafish	1.25–5 mg/L	Activate the Nrf2 pathway.	[81]
	10–20 mg/L	Inhibit the Nrf2 pathway, leading to oxidative stress, inflammation, and apoptosis in zebrafish liver tissue.	
	2–4 μg/L	48 h after fertilization, the survival rate of zebrafish embryos decreased significantly.	[87]
Male lizard	40 mg/kg	The lizards’ testicles sustained oxidative damage, and the activity of glutamine synthetase in their livers was severely inhibited.	[84]
Earthworm	0.4–250 mg/kg	Body weight, cocoons, and larvae were significantly reduced, and as the exposure time increased, DNA damage in the coelomic cells of earthworms gradually worsened.	[88]
*Microcystis aeruginosa*	0.5 and 1 mg/L	Promote the growth of *M. aeruginosa*.	[77]
	5 and 10 mg/L	Induce MDA levels in *M. aeruginosa*, and inhibit the growth of *M. aeruginosa*.	
*Daphnia magna*	EC_50_: 1193 ± 89.2 mg/L	At 48 h, the mortality rate of *D. magna* reached 50%.	[89]

## Data Availability

No new data were created or analyzed in this study. Data sharing is not applicable to this article.
